# Identification of an Immune Gene-Based Cisplatin Response Model and CD27 as a Therapeutic Target against Cisplatin Resistance for Ovarian Cancer

**DOI:** 10.1155/2022/4379216

**Published:** 2022-05-18

**Authors:** Ying Guo, Na Yang, Gang Li, Xiurong Yin, Lixia Dong

**Affiliations:** Department of Obstetrics and Gynecology, Maternity and Child Health Hospital of Qinhuangdao, Qinhuangdao, 066000 Hebei, China

## Abstract

**Objective:**

Evidence demonstrates that the immune microenvironment is extensively associated with chemotherapy response of ovarian cancer (OV). Herein, this study is aimed at establishing a cisplatin response prediction model for OV on the basis of immune genes.

**Methods:**

The expression profiles of cisplatin-sensitive and cisplatin-resistant OV specimens were integrated from multiple public datasets. The abundance scores of 22 immune cells were estimated with CIBERSORT algorithm. Differentially expressed immune genes (DEGs) were determined between cisplatin-sensitive and cisplatin-resistant groups. Thereafter, a cisplatin response model was constructed based on prognostic DEGs with logistic regression analysis. The prediction performance was validated in independent cohorts. The possible relationships between the model and immunotherapy were then assessed.

**Results:**

Treg scores were significantly decreased in cisplatin-resistant than cisplatin-sensitive OV specimens, with the opposite results for naïve B cells and activated dendritic cells. Fourteen prognostic DEGs were identified and used to develop a cisplatin-response model. The response scores, estimated by the model, showed favorable performance in discriminating cisplatin-response and nonresponse samples. The response scores also presented significantly negative correlations with three well-known cisplatin-resistant pathways and a positive correlation with the expression of CD274 (PD-L1). Moreover, the decreased CD27 expression was observed in cisplatin-resistant groups, and OV specimens with higher CD27 expressions were more sensitive to cisplatin treatment.

**Conclusion:**

Altogether, our findings proposed a cisplatin response prediction model and identified CD27 that might be involved in cisplatin resistance. Further investigations suggested that CD27 could be a promising immunotherapeutic target for cisplatin-resistant subset of OV.

## 1. Introduction

Ovarian cancer (OV) is one of the most malignant gynecologic tumors that are difficult to cure [1]. Cisplatin is the first-line platinum-based chemotherapeutic drug of OV. Most patients initially show response to chemotherapy, but more than 70% will develop recurrent disease in three years [2]. Chemoresistance limits the survival of patients with advanced cancer who receive chemotherapy [3]. Nevertheless, the genome-wide alterations in gene expressions are not well understood during the transition from chemosensitivity to chemoresistance. Identifying targets with clinical translation potential is still an unsolved challenge. Recent evidence suggests that epigenetic variations and multiple pathways contribute to chemotherapy resistance [4–6]. It has been reported that changes in cellular drug efflux, elevated cellular glutathione levels, increased DNA repair, and drug tolerance are associated with the acquisition of platinum resistance [7, 8]. Although ATP-binding cassette (ABC) transporters are key proteins for multiple drug resistance, a resistance phenotype is still observed in the expression absence of some drug resistance genes [9], suggesting the complex principles of drug resistance in cancer cells and the possible involvement of many unknown biological pathways. BCL2-associated agonist of cell death (BAD) family, a BH3-only member of the Bcl-2 family, exerts a key function in modulating apoptosis [10]. Phosphorylation of this pathway is associated with cisplatin resistance in OV and patients' outcome [11]. Moreover, extracellular matrix (ECM) has been reported to correlate with drug resistance, which is commonly recognized by inhibiting drug penetration into the cancer tissues and the interaction between ECM components and cancer cells [12].

Immunotherapy enhances the antitumor immune response in a variety of ways, one of which is through monoclonal antibodies that act on immunosuppressive ligands expressed on tumor cells, also known as immune checkpoint inhibitors (ICIs) [13–15]. With the assistance of immune checkpoints, T lymphocytes are able to discriminate their own cells from heterologous cells [16, 17]. Immune evasion occurs when cancer cells upregulate the expression of immune checkpoints to facilitate their escape from the recognition of T cells [18]. ICIs block the immune escape of cancer cells by binding to CTLA-4, PD-1 or PD-L1, etc., thereby recovering the killing activity of T cells against cancer cells [19–21]. Regrettably, a recent clinical trial showed that combination of PD-L1 antibody atezolizumab and platinum-based chemotherapy and bevacizumab did not significantly improve the overall survival of newly diagnosed stage III or IV OV [22]. Increasing evidence demonstrates that both the type and extent of immune infiltration correlate with OV patients' outcome [23]. In a recent clinical trial, the addition of dendritic cell-based immunotherapy to the first-line chemotherapy (carboplatin plus paclitaxel) significantly improved progression-free survival of epithelial OV patients [24]. Resolution of immune cells in the exudate of OV patients revealed that higher proportions of B cells and NK cells corresponded to worse patient survival, with the coincidence of higher B cells in FIGO stage IV patients than in stage III [25]. Cytotoxic CD8+ tumor-infiltrating lymphocytes (TILs) are also involved in immune regulation of OV [26]. In a cohort study with over 5000 cases, increased CD8+ TILs were accompanied by improved survival in OV patients, and this log-linear relationship was independent of postoperative residual and germline BRCA1 mutations [26].

Despite the proposal of a variety of novel cancer treatments represented by cytotoxic anticancer drugs and target therapies, the control of OV progression remains inadequate [27]. The low efficacy of chemotherapy mainly attributes to the developed drug resistance of cancer cells, revealing that the underling molecular mechanism related to chemoresistance is a crucial step to improve patients' survival. New therapeutic paradigms are urgently needed to overcome drug resistance in OV. The availability of immunotherapy offers a promising pathway for the treatment of OV. However, patients usually show selective response to immunotherapy, thereby limiting its application in clinical practice. Based on these key issues, we attempted to construct a cisplatin response prediction model on the basis of prognostic immune genes, which will facilitate the better application of immunotherapy on the cisplatin-resistant subset of OVs.

## 2. Materials and Methods

### 2.1. Study Design and Data Preprocessing

Five datasets including The Cancer Genome Atlas-OV (TCGA-OV) cohort and 4 eligible gene expression profiles from the Gene Expression Omnibus (GEO) database were downloaded to investigate the alterations of immune microenvironment related to cisplatin response. The design of this study is shown in Supplementary figure 1. The four GEO datasets were selected because they consisted of the gene expression profiles of both cisplatin-resistant and cisplatin-sensitive samples after cisplatin treatment. The 547 immune genes were extracted from the 22 immune cell-related genes provided by Cell type Identification By Estimating Relative Subsets Of RNA Transcripts (CIBERSORT) [28]. The GSE23553 dataset contained gene expression profiles of 6 paired samples of cisplatin-resistant and cisplatin-sensitive A2780 cell lines [11], which was used to investigate differentially expressed immune genes (DEGs) between resistant and sensitive groups. TCGA-OV cohort composed of 373 samples with expression profiles was used to identify prognostic DEGs. In addition, a cisplatin response model was developed based on TCGA-OV dataset that included 209 complete response and 27 progressed disease samples after chemotherapy, which was validated in the GSE156699 dataset consisting of 38 cisplatin nonresponse patients and 50 response patients [29]. Possible associations of the model with other cisplatin resistance pathways as well as immune checkpoint genes were also investigated in TCGA-OV dataset. The single-cell expression profiles of six OV patients who received chemotherapy in the GSE158722 dataset [30] were used to evaluate the variations of response scores with chemotherapy proceeding. Both the GSE148392 [31] and GSE23554 [11] datasets included OV tissue samples that showed resistance or sensitivity to cisplatin, which were used to verify the expression patterns of CD27 on tumor tissues. Finally, we investigated the relationship between CD27 expression and cisplatin response in 19 OV cell lines provided by the Genomics of Drug Sensitivity in Cancer (GDSC) database [32].

### 2.2. Estimation the Abundance Scores of 22 Immune Cells

The CIBERSORT tool was used to estimate the abundance scores of 22 immune cells for 12 samples in the GSE23553 dataset. The LM22 matrix contained expression profiles of 547 immune genes that represented the expression patterns of 22 immune cell-associated genes, which was used as the background input of CIBERSORT. The perturbation was set as 1000 times with other parameters default.

### 2.3. Differential Expression Analysis

Nonparametric rank sum test was performed to assess the variations of 22 immune cell scores as well as the expression profiles of 547 immune genes between cisplatin-resistant and cisplatin-sensitive groups. The significantly different immune cells and immune genes were determined with a *p* value less than 0.05.

### 2.4. Prognostic Analysis

TCGA-OV dataset which included the patients' prognostic information, was selected for survival analysis. After excluding the samples without prognostic information and the survival time less than 30 days, a total of 373 samples were retained to identify prognostic DEGs. The “coxph” method in “survival” package was used to evaluate the relationship of gene expression with survival time and events. DEGs with a log-rank test *p* value less than 0.05 were defined as prognostic genes. In addition, the hazard ratio and its 95% confidence interval were also estimated.

### 2.5. Developing the Cisplatin Response Model

Fourteen prognostic DEGs were identified and used to develop a cisplatin response model for OV. TCGA-OV and GSE156699 datasets were chosen as the training and test sets, as they both contained sufficient cisplatin response and nonresponse samples (236 and 88 samples, respectively). Logistic regression was adopted to fit the model, and the cisplatin response scores of all samples were subsequently calculated. Receiver operator characteristic curve (ROC) analysis was conducted to assess the performance of this model, and the area under the curve (AUC) was also calculated. The optimal sensitivity and specificity were estimated when Youden index reached the maximum.

### 2.6. Cisplatin Response Analysis

The drug response data of OV cell lines were retrieved from the GDSC database (https://cancerrxgene.org/). The half-maximal inhibitory concentration (IC50) value of cisplatin and gene expression profiles of 19 OV cell lines were used to explore the relationship of cisplatin response with CD27 expression. The Spearman rho statistic and the corresponding *p* value were calculated to estimate the correlation.

### 2.7. Statistical Analysis

Data analysis and figure preparation were conducted by R software (version 4.1). The comparisons of continuous variables between two groups were performed using Wilcoxon test, while multiple comparisons were conducted using Kruskal test. For the comparisons of categorical variables, chi-squared contingency table tests (if the counts of all categories were not less than 5) or Fisher's exact tests (if the count of one category was less than 5) were performed. The symbols “ns,” “∗,” “∗∗,” “∗∗∗,” and “∗∗∗∗” indicated *p* value > 0.05, ≤0.05, ≤0.01, ≤0.001, and ≤0.0001, respectively.

## 3. Results

### 3.1. Characterization of Immune Microenvironment Differences between Cisplatin-Sensitive and Cisplatin-Resistant OVs

**Figure 1:**
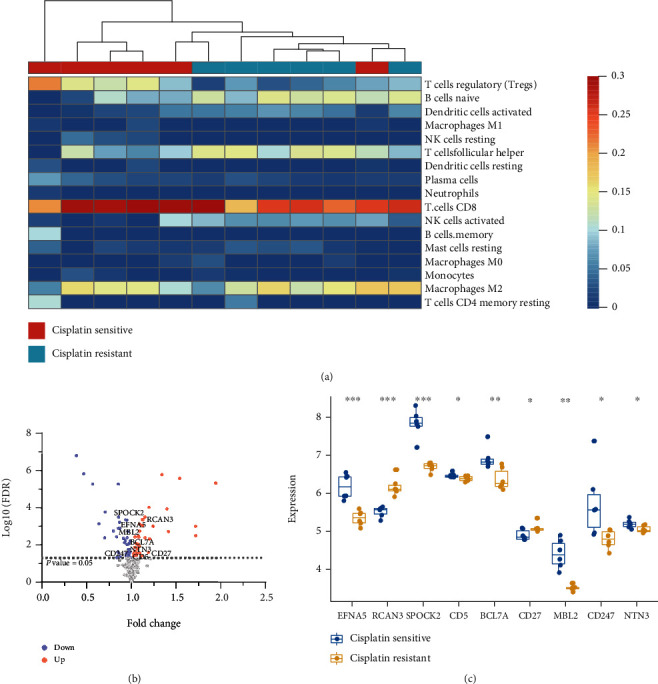
The immune microenvironment of cisplatin-resistant and cisplatin-sensitive OV groups. (a) The immune cell scores of cisplatin-resistant and cisplatin-sensitive groups. The dendrogram showed the hierarchical clustering results of cisplatin-resistant and cisplatin-sensitive groups. (b) Differentially expressed immune genes between cisplatin-resistant and cisplatin-sensitive groups. The red and blue points represented upregulated and downregulated genes, respectively. The texts indicated the symbols of nine Treg-related genes. (c) The expression profiles of nine Treg-related genes in cisplatin-resistant and cisplatin-sensitive groups.

The abundance scores of three immune cells showed significant differences in cisplatin-resistant and cisplatin-sensitive cell lines, including T cells regulatory (Tregs), B cells naïve, and dendritic cells activated (Supplementary table 1, *p* value < 0.01). Tregs of the resistant group presented lower scores than those of the sensitive group, with the opposite results for B cells naïve and dendritic cells activated (Figure 1(a)). We identified 86 DEGs between cisplatin-resistant and cisplatin-sensitive groups, of which 37 were upregulated and 49 were downregulated in the resistant group (Supplementary table 2, Figure 1(b)). Nine DEGs were related to Tregs according to the LM22 matrix, including two upregulated genes, CD27 and RCAN3, and seven downregulated genes, BCL7A, CD247, CD5, EFNA5, MBL2, NTN3, and SPOCK2 in the resistant group (Figure 1(c)).

### 3.2. Identification of Prognostic DEGs

**Figure 2:**
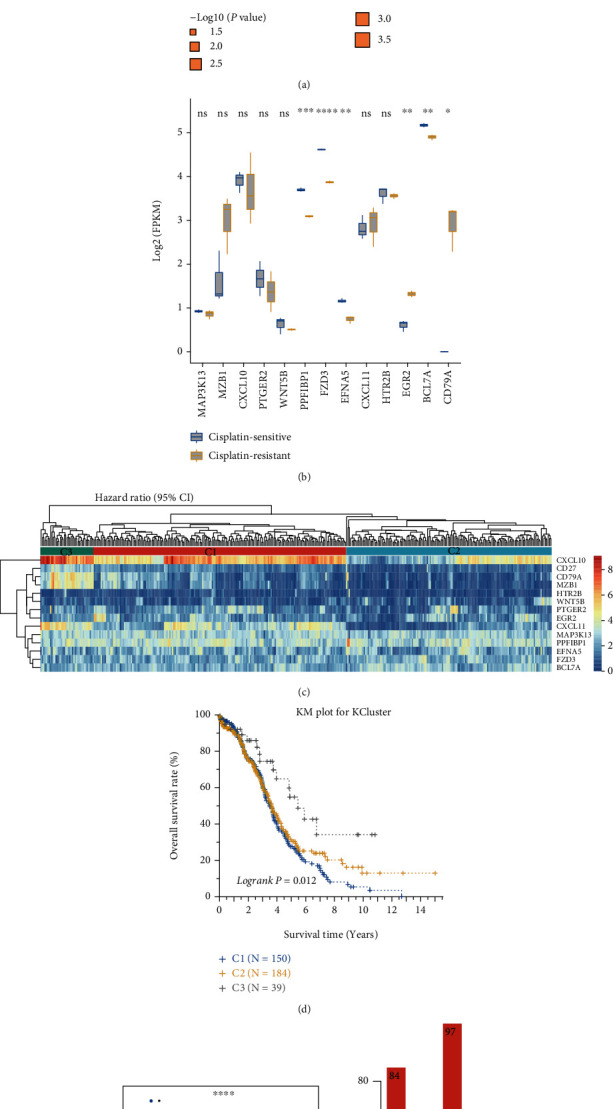
Prognostic analysis of differentially expressed immune genes. (a) Hazard ratios of the 14 prognostic DEGs. The rectangle size represented the -log10 of *p* value. CI: confidence interval. (b) The expression values of 13 prognostic DEGs validated in the GSE173201 dataset. (c) The expression profiles of 14 prognostic DEGs in TCGA-OV dataset. The up dendrogram showed the hierarchical clustering results of TCGA-OV samples. The up side bars indicated the three groups C1, C2, and C3. (d) Survival curves showed the overall survival rate of the samples between three groups. (e) The Treg scores of the samples between three groups. (f) The distributions of CR and PD samples between the three groups. CR: chemotherapy response; PD: disease progressed.

Survival analysis identified that 14 DEGs were associated with OV prognosis (log rank *p* value < 0.05, Figure 2(a)), of which 6 were favorable prognostic genes (hazard ratio < 1) and 8 were poor prognostic genes (hazard ratio > 1). The GSE173201 dataset, which consisted of 3 cisplatin-resistant and cisplatin-sensitive samples, was used to verify the 14 DEGs. We observed significant differences for more than half of the 13 DEGs (*n* = 7, no expression values were available for CD27; Figure 2(b)). Based on the expression profiles of the 14 genes, samples in TCGA-OV cohort were clustered into 3 groups, C1 (*n* = 150), C2 (*n* = 184), and C3 (*n* = 39) (Figure 2(c)). Five genes, CXCL10, CD27, CD79A, MZB1, and CXCL11, exhibited the highest expression values in the C3 group, which were mainly associated with T cells, B cells, and dendritic cells (Supplementary figure 2). Survival analysis revealed that samples of the C3 group harbored the best prognosis. The overall survival rate of the C2 group samples showed a little better than C1 but no significant variation was observed (Figure 2(d)). Interestingly, Treg scores showed a decreasing trend across the three groups with C3 presenting the highest, followed by C2, and the lowest on C1 (Figure 2(e), *p* value < 0.001). However, no significant variations were found for B cells naïve and dendritic cells activated (Supplementary figure 3). In addition, the C3 group was dominated by the chemotherapy-response samples (96.55%), while the disease-progressive samples were almost absent (Figure 2(f), 3.45%).

### 3.3. Developing a Cisplatin Response Model for OV

**Figure 3:**
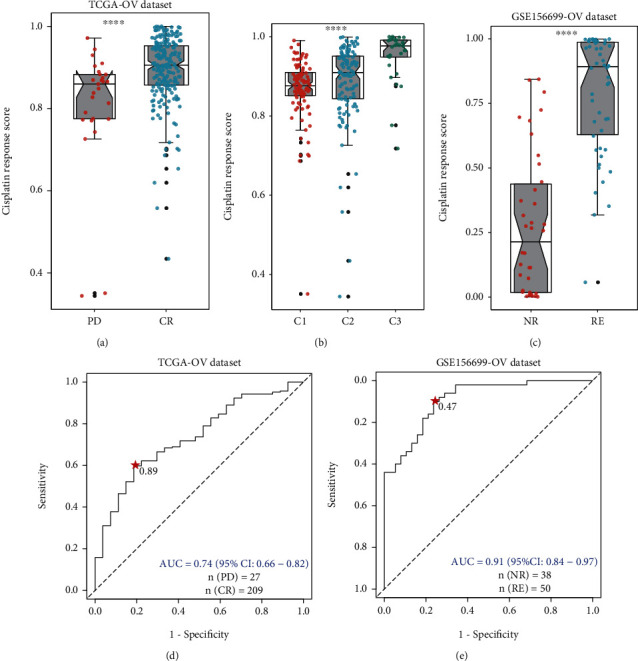
The performance of the cisplatin response model. (a) The cisplatin response scores of CR and PD samples in TCGA-OV dataset. PD: disease progressed; CR: complete response. (b) The cisplatin response scores of samples between the three groups. (c) The cisplatin response scores of RE and NR group samples in the GSE156699 dataset. NR: nonresponse; RE: response. (d) ROC curve showed the performance of the cisplatin response model in TCGA-OV dataset. (e) ROC curve showed the performance of the cisplatin response model in the GSE156699 dataset. The red stars indicated the best cut-off values when Youden's index reached the maximum in (d) and (e).

**Figure 4:**
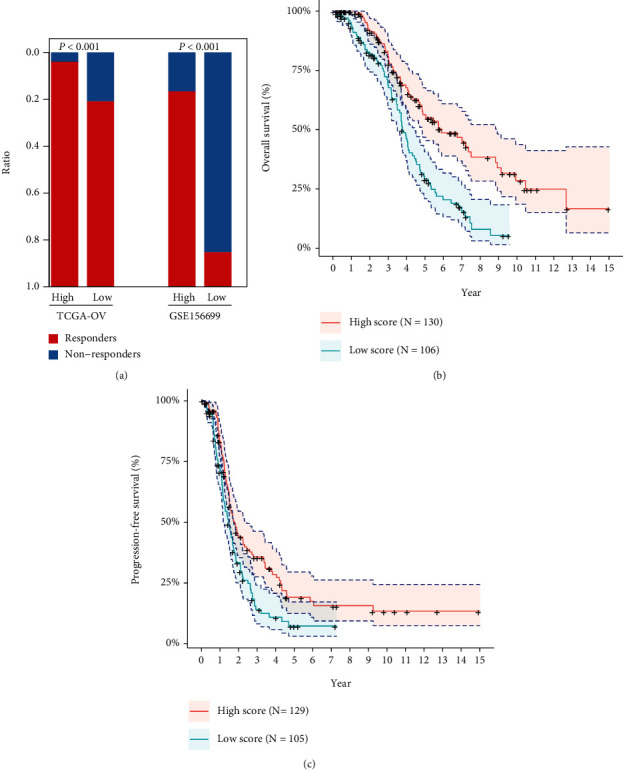
Prognostic analysis of OV samples between high- and low-score groups. (a) The distributions of response and nonresponse samples between high- and low-score groups. (b, c) The overall survival rate and progression-free survival rate of high- and low-score group samples in TCGA-OV dataset. The cross symbols indicated censored samples. The 95% confidence intervals were also presented in (b) and (c).

According to the patients' chemotherapy outcomes provided by TCGA-OV dataset, samples of complete response (CR) and disease progressed (PD) groups were retained and defined as cisplatin-sensitive and cisplatin-resistant phenotypes, respectively. Fourteen prognostic DEGs were used to construct the logistic regression model for cisplatin response. The response scores were subsequently estimated for all samples, which represented the probability of one sample being classified as PD. The response scores of CR samples were significantly higher than those of PD samples (Figure 3(a)). The scores of C1, C2, and C3 also presented an increased trend (Figure 3(b)). The response group samples in the test set also showed higher scores than nonresponse group samples (Figure 3(c)). ROC curve analysis was conducted to evaluate the performance of the model to distinguish CR from PD samples. The AUC was 0.74 (95% CI: 0.63-0.81). The optimal specificity and sensitivity were 81.5% and 59.8%, respectively, when the score threshold equaled 0.89 at the maximized Youden index (Figure 3(d)). The AUC value was 0.80 with the optimal sensitivity and specificity of 68.0% and 81.6% at the threshold of 0.65 in the test set (Figure 3(e)). Samples in the two datasets were divided into a high-score group (H-score) and low-score group (L-score) according to their respective thresholds, and the majority of nonresponse samples were distributed in the H-score group (Figure 4(a)). H-score samples also showed worse overall survival and progression-free survival than L-score samples (log rank *p* values < 0.001, Figures 4(b) and 4(c)).

### 3.4. Association of Cisplatin Response Model with Chemotherapy-Resistant Pathways

**Figure 5:**
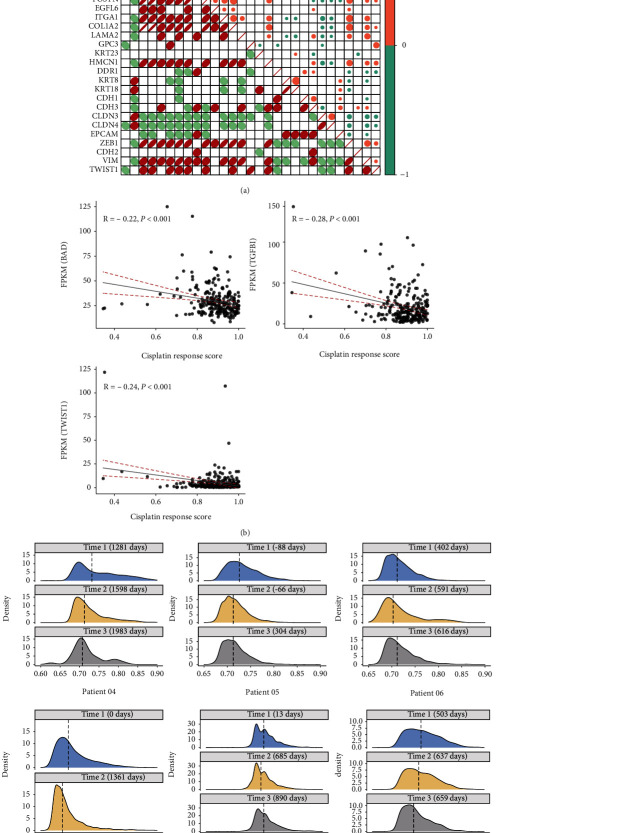
The association of cisplatin response scores with resistant pathways. (a) The correlation matrix between cisplatin response scores and the expression levels of genes in BAD apoptosis, ECM, and EMT pathways. The red and green colors indicated positive and negative correlations. The circle size represented the correlation coefficient. Cscore: cisplatin response score. (b) The correlation of cisplatin response scores with the expression values of BAD, TGFB, and TWIST1. (c) The density of cisplatin response scores between the cells of three time points in six patients. The *x*-axis was the cisplatin response scores, and the dashed line showed the median response score.

Previous studies have revealed that the emergence of cisplatin resistance in OV is associated with the alterations of multiple pathways, including the BAD apoptotic pathway, ECM, and epithelial to mesenchymal transition (EMT). We obtained 30 genes related to these pathways, of which 28 gene expressions can be detected, including 1 BAD pathway gene, 15 ECM pathway genes, and 12 EMT-related genes according to the references [11, 33]. Correlation analysis revealed that cisplatin response scores showed significantly negative correlations with six genes (Figure 5(a)). Although not all genes exhibited strong correlations with cisplatin response scores, three key genes of these pathways, BAD (BAD apoptotic pathway), TGFB (ECM pathway), and TWIST1 (EMT pathway), negatively correlated with the response scores (Figure 5(b)). We attempted to evaluate the alterations of cisplatin response scores among OV patients receiving chemotherapy after surgery in the GSE158722 dataset that consisted of single-cell expression profiles of 17 samples collected from 6 patients at three time points. The cisplatin response scores presented a decreasing trend with the treatment time increased for patient 04, patient 05, patient 07, and patient 09 (Figure 5(c)), and the differences between three time points were significant (Supplementary figure 4). The decreased response scores suggested that some tumor cells might obtain cisplatin resistance with the treatment proceeded.

### 3.5. Association of Cisplatin Response Model with Immune Checkpoints

**Figure 6:**
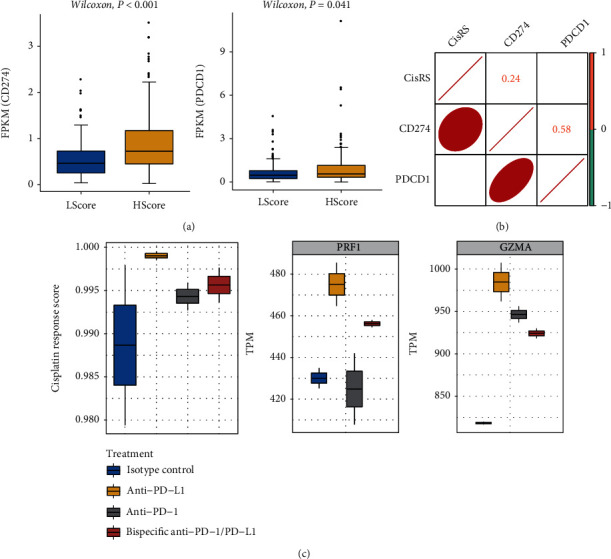
The association of cisplatin response scores with immunotherapy. (a) The expression values of CD274 and PDCD1 in high-score (HScore) and low-score (LScore) groups. FPKM: fragments per kilobase per million. (b) The correlation matrix showed the correlations between cisplatin response scores (CisRS) and CD274 and PDCD1. (c) The cisplatin response scores and expression values of PRF1 and GZMA between the four groups, isotype control, anti-PD-L1 treated, anti-PD-1 treated, and bispecific anti-PD-1/PD-L1 treated. TPM: transcripts per million.

In recent years, the immune checkpoint inhibitors have been widely investigated as promising drugs for immunotherapy. We then explored the potential association of cisplatin response scores with the immune checkpoint genes. The expression values of PDCD1 (PD1) and CD274 (PD-L1) were significantly elevated in the high-score group in TCGA-OV dataset (Figure 6(a)). The response scores also significantly correlated with CD274 expression, but not with PDCD1 expression (Figure 6(b)). The relationship between cisplatin response scores and CD274 was further verified in the GSE160752 dataset, which provided the expression profiles of 16 OV samples receiving anti-PD-1 and anti-PD-L1 treatments. The samples that received anti-PD-L1 treatment showed the highest response scores, but no significance was obtained due to the limited samples (Figure 6(c)). Moreover, the two genes, PRF1 and GZMA, act synergistically to exert immune cytolytic activity, an essential component of adaptive immunity, which also exhibited the highest expression levels in anti-PD-L1-treated samples (Figure 6(c)), indicating the increased immune activation.

### 3.6. Association of CD27 with Cisplatin Response

**Figure 7:**
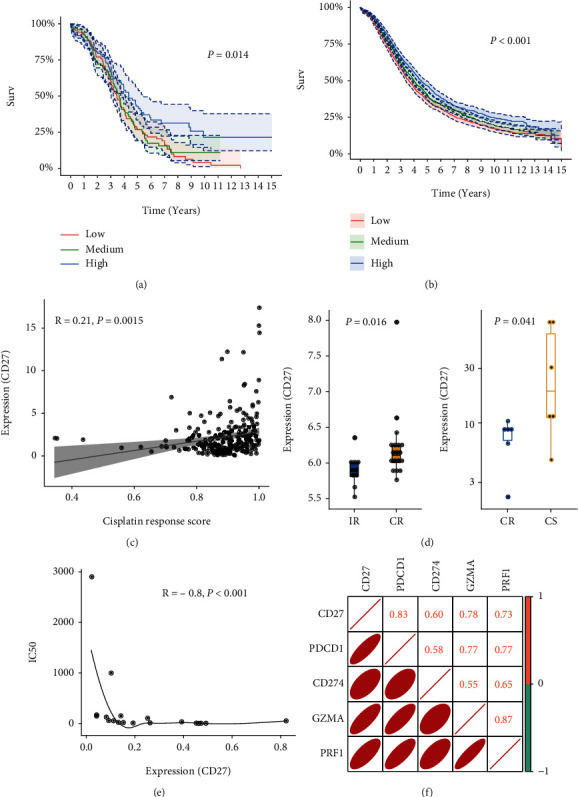
The association of CD27 expression with cisplatin response. (a, b) KMplot showed the overall survival rates of high-, medium-, and low-expression groups in TCGA and GSE135820 datasets. (c) The correlation of CD27 expression with cisplatin response scores. The Spearman rho statistic and the corresponding *p* value were estimated. (d) The expression values of CD27 between cisplatin response and nonresponse groups in the GSE23554 (left) and GSE148392 (right) datasets. (e) The correlation of CD27 expression with IC50 values on 19 OV cell lines. The Spearman rho statistic and the corresponding *p* value were estimated. (f) The correlation matrix showed the correlations of CD27 expression with the four genes, PDCD1, CD274, GZMA, and PRF1.

Survival analysis indicated that high Treg scores were associated with better prognosis of OV, and its marker gene CD27 also showed a positive correlation with good prognosis of OV. These findings suggested that CD27 might play a pivotal role in altering the response of OV cells to cisplatin treatment. Therefore, we focused on this gene for further analysis. According to the expression level of CD27, TCGA-OV samples were divided into high-expression, moderate-expression, and low-expression groups. Samples of the high-expression group showed a better overall survival rate than those of the other two groups (Figure 7(a)), which was validated in the GSE135820 dataset (Figure 7(b)). The expression of CD27 also positively correlated with cisplatin response scores (Figure 7(c)). The gene expression profiles of the GSE23554 and GSE148392 datasets that consisted of 18 and 12 OV tissue samples after cisplatin treatment were retrieved from the GEO database to verify the relationship of CD27 expression with cisplatin treatment. The CD27 expressions were significantly downregulated in cisplatin nonresponse groups in both datasets (Figure 7(d)). Moreover, we observed a negative correlation of CD27 expression with the IC50 values in 19 OV cell lines (Figure 7(e)). In addition, the expression of CD27 with other four genes, PDCD1, CD274, GZMA ,and PRF1, also presented strong correlations (Figure 7(f)).

## 4. Discussion

OV is one of the most common malignant tumors in women, and chemotherapy resistance is a key factor leading to the high mortality of this disease [34]. Therefore, exploring the feasibility of immunotherapy for chemotherapy-resistant individuals would be an important and valuable work. The current study investigated the alterations of immune microenvironment between cisplatin-resistant and cisplatin-sensitive OV cells and developed a cisplatin response model using 14 prognostic immune genes. The model showed a strong correlation with the immune checkpoint gene CD274 (PD-L1). Furthermore, we found that CD27 might be a potential marker for OV patients.

Tregs have been reported to be decreased in OV samples treated by neoadjuvant chemotherapy [35], which is consistent with the observations of this study that Treg scores were downregulated in the cisplatin-resistant group. Naïve B cells and activated dendritic cells showed the opposite trend, implying that their alterations might be associated with the response of OV cells to cisplatin. The findings of this study indicated that the scores of Tregs were the highest for the C3 group that had the best prognosis and were the lowest for the C1 group, suggesting that Tregs might be a good prognostic factor of OV. However, in the past time, studies about the impact of Tregs on OV clinical outcome have exhibited apparently conflicting results. The study of Curiel et al. creatively demonstrated that intratumoral Tregs were associated with low survival rate of OV [36]. An inverse relationship between the density of intratumoral Tregs and the overall survival rate of OV was also reported in several studies [37, 38]. Besides, no relationship between Tregs and patient outcome was observed in a study [39]. These contradictory data may be interpreted from several aspects. Firstly, it may be linked to tumor heterogeneity [40]. Secondly, the imperfect markers of phenotypical cells directly lead to some immune cells missed by CIBERSORT. Third, in terms of drug response, this inconsistency may reflect remodeling of the tumor microenvironment due to the transition of tumor cells from cisplatin sensitive to resistant [35].

Primary or acquired chemoresistance is a key determinant for the high mortality rate of OV [34]. Accurate assessment of chemotherapy response is one of the effective approaches to address this issue. For chemotherapeutic agents, cisplatin-induced DNA damage is the main therapeutic mechanism. Therefore, a cisplatin response model based on the DNA damage repair genes has been chosen to provide this predicted prognostic information [41]. In addition, methylation CpGs and miR-206 have been used to construct predictive models and both showed good performance [4, 42]. Several chemotherapy response prediction models have been proposed. For instance, an immune cell infiltration score was developed that enabled to predict the sensitivity to cisplatin for OV patients [43]. An integrated analysis of gene expression profiling proposed that an 18-gene model predicted the response of platinum/paclitaxel-based treatment in epithelial OV [44]. Based on digital immune-related gene signatures, a platinum response prediction model was established for OV [45]. However, none of them have been applied in clinical practice. Growing evidence has demonstrated the tight linkage between the immune response and platinum response, involving the immune microenvironment remodeling during the transition from cisplatin sensitivity to resistance [46]. Nevertheless, cisplatin response models based on immune genes are rarely reported. The current study presented a cisplatin response model based on 14 immune-related genes, which showed well-performed discrimination between cisplatin-sensitive and cisplatin-resistant samples both in the training and test sets. Moreover, OV patients with high response scores also harbored better overall survival and progression-free survival rates than those with low scores. These results indicated that the cisplatin response model showed the potential to predict the response of OV patients to cisplatin treatment, which may help physicians to assess the cisplatin-sensitive or cisplatin-resistant patients before they received chemotherapy.

Our cisplatin response model consisted of CD27, RCAN3, BCL7A, CD247, CD5, EFNA5, MBL2, NTN3, and SPOCK2. Limited evidence demonstrates the roles of the above genes in OV progression. For instance, a prime/boost vaccine platform identified CD27 agonist and loss of myeloid-derived suppressor cells as treatment that combined with ICIs in OV [47]. Low BCL7A expression predicted undesirable survival outcome of OV patients [48]. CD247 expression was linked to differentiation and classification in OV [49]. EFNA5 was upregulated in aggressive and recurrent OV [50]. Binding to DNA is the basis for the cytotoxicity of cisplatin on tumor cells. The three well-known mechanisms of intracellular chemoresistance comprise reduction of drug cell accumulation, drug intracellular detoxification, and DNA damage repair [51]. Although they have been recognized as the key pathways for cisplatin resistance, several novel pathways have been found, including the BAD apoptotic pathway, ECM, and EMT. Cisplatin resistance is a complex biological phenomenon associated not only with adaptive mechanisms within tumor cells but also with the microenvironment outside the tumor cells. We found that the cisplatin response scores showed significantly negative correlations with the three pathways, suggesting the close connections between them.

Previous knowledge suggests that chemotherapeutic agents induce immunosuppression; however, recent studies have highlighted the critical impact of tumor microenvironment on cisplatin resistance [52]. In OV murine models, cisplatin delivery increased the expression of immune checkpoint receptor PD-L1, indicating a positive impact of cisplatin treatment on immune response [53]. In this study, PD-L1 expression was upregulated in the high-score group and negatively correlated with cisplatin response scores, indicating that the immune response was perturbed after cisplatin treatment. Indeed, the tumor cytotoxicity could be regulated by cisplatin through modulating cytotoxic effectors and suppressing immune cells within tumor microenvironment, thereby exerting positive anticancer effects [54]. Our findings also implied that cisplatin resistance may be associated with low PD-L1 (CD274) expression.

CD27, a member of tumor necrosis factor receptor superfamily (TNFRSF), is expressed on a wide range of human lymphocytes such as memory T cells and Tregs. It acts as a costimulatory receptor through collaboration with its natural ligand CD70 to activate T cells [55]. Evidence demonstrates that CD27 plays a pivotal role in enhancing the generation and maintenance of antigen-specific CD4+ and CD8+ T cells [56]. In this study, we found that CD27 expression was significantly downregulated in the cisplatin-resistant group and negatively correlated with sensitivity of OV cell lines to cisplatin. These findings revealed that CD27 expression was suppressed on cisplatin-resistant samples, suggesting a failed T cell (CD4+ or CD8+) activation.

Recent years have witnessed the emergence of immunotherapy based on immune checkpoint genes, but not all patients show a positive response due to insufficient T cell initiation. Accumulating evidence suggests that CD27 agonist antibodies exhibit specific synergistic effects with CTLA-4 and PD-1 inhibitors, especially when combined with PD-1 inhibitors; the strategy successfully eliminated tumors in preclinical models [57]. The synergistic antitumor activity of PD-1 inhibitors with CD27 agonist antibodies, varlilumab, was also observed on progressed OV patients. The patients who received combination therapy had better outcomes and did not obtain additional toxicity [58]. Our findings indicated that suppressed expression of CD27 may be associated with the cisplatin-resistant phenotype of OV, providing an alternative therapeutic target for the subset of cisplatin-resistant patients. The combination with ICIs might be an effective strategy to improve the prognosis of cisplatin-resistant patients. However, activation of the CD27/CD70 axis may also lead to tumor immunosuppressive effects by enhancing the survival of natural Tregs and inducing the apoptosis of effector T cells [59, 60]. Therefore, more evidence is required for CD27 as a therapeutic target for the chemoresistant patients.

However, several limitations should be pointed out. Our study was based on the data from public datasets. The fundamental research was essential to explain the internal mechanisms of our cisplatin response model. Moreover, clinical studies are still required for validating the predictive value of this cisplatin response model.

## 5. Conclusion

Altogether, our findings proposed a reliable cisplatin response model on the basis of 14 immune genes for OV patients. Additionally, CD27, a costimulator of activated T cells, was recognized as a favorable prognostic factor, and its expression showed significant downregulation in cisplatin-resistant groups, which might be a promising candidate immunotherapeutic target for the subset of cisplatin-resistant patients.

## Data Availability

The data used to support the findings of this study are included within the supplementary information files.

## Abbreviations

DEGs: Differentially expressed immune genes
OV: Ovarian cancer
ABC: ATP-binding cassette
BAD: BCL2-associated agonist of cell death
ECM: Extracellular matrix
ICIs: Immune checkpoint inhibitors
TILs: Tumor-infiltrating lymphocytes
TCGA: The Cancer Genome Atlas-ovarian cancer
GEO: Gene Expression Omnibus
CIBERSORT: Cell type Identification By Estimating Relative Subsets Of RNA Transcripts
GDSC: Genomics of Drug Sensitivity in Cancer
ROC: Receiver operator characteristic curve
AUC: Area under the curve
IC50: Half-maximal inhibitory concentration
Tregs: T cells regulatory
CR: Complete response
PD: Disease progressed
EMT: Epithelial to mesenchymal transition.

## Supplementary Materials

Supplementary 1 Supplementary Figure 1: the flowchart of this study. OV: ovarian cancer; EMT: epithelial to mesenchymal transition; ECM: extracellular matrix; IC50: half-maximal inhibitory concentration.

Supplementary 2 Supplementary Figure 2: upset plot showing the relationships of 14 immune genes with 22 immune cell types.

Supplementary 3 Supplementary Figure 3: the scores of naïve B cells and activated dendritic cells between the three groups. Ns: not significant.

Supplementary 4 Supplementary Figure 4: the cisplatin response scores at three time points for patient 04 (A), patient 05 (B), patient 06 (C), patient 07 (D), patient 08 (E), and patient 09 (F).

Supplementary 5 Supplementary Table 1: the scores of LM22 cell types between the cisplatin-sensitive (A2780S) and cisplatin-resistant (A2780CP) groups in the GSE23553 dataset.

Supplementary 6 Supplementary Table 2: eighty-six DEGs between cisplatin-resistant and cisplatin-sensitive groups.
