# Supplementary figures and images for: Identification of an Immune Gene-Based Cisplatin Response Model and CD27 as a Therapeutic Target against Cisplatin Resistance for Ovarian Cancer
Source: J Immunol Res. 2022 May 18;2022:4379216. doi: 10.1155/2022/4379216 (PMC9133897; doi:10.1155/2022/4379216)

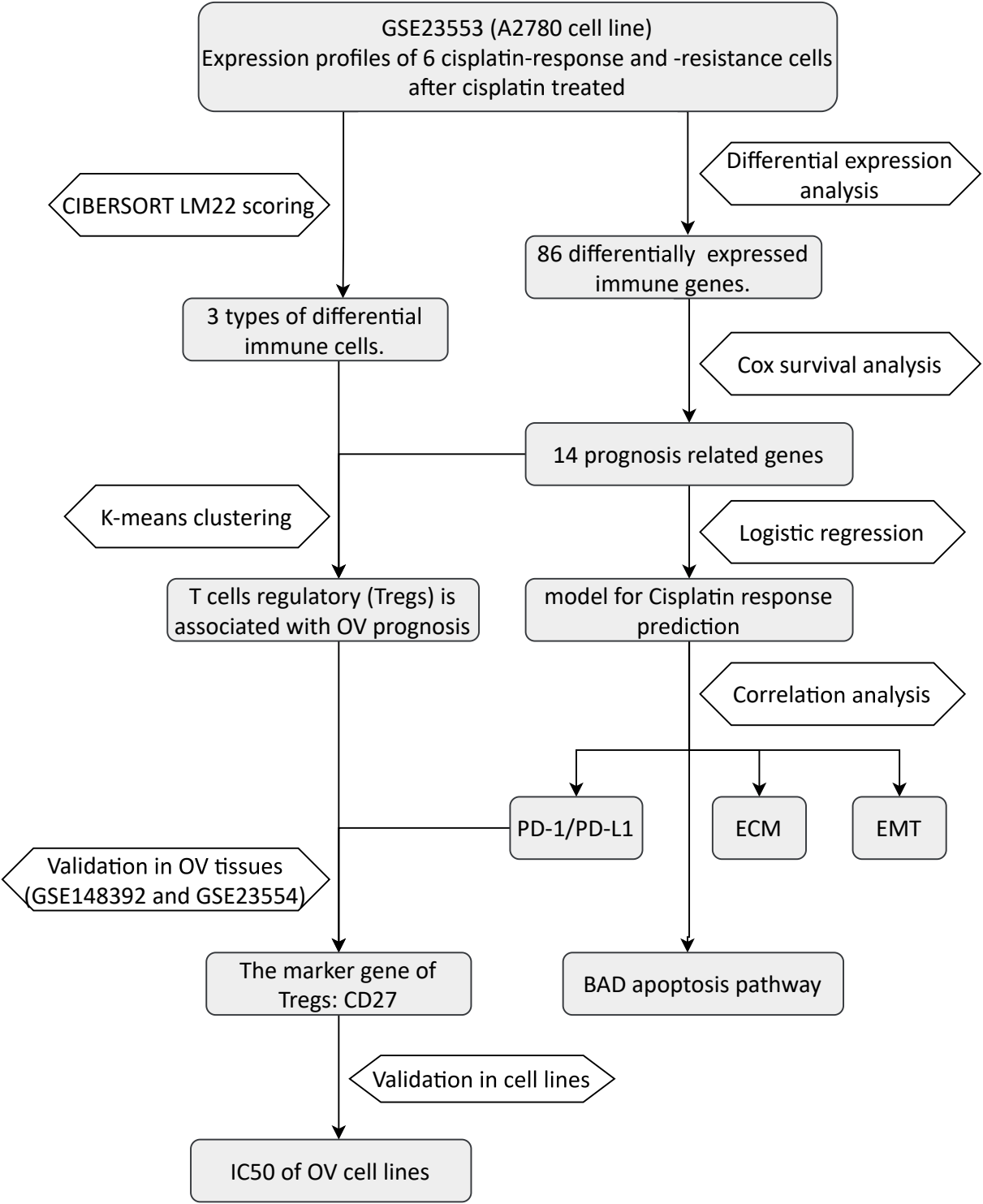

Supplement: Supplementary 1 — Supplementary Figure 1: the flowchart of this study. OV: ovarian cancer; EMT: epithelial to mesenchymal transition; ECM: extracellular matrix; IC50: half-maximal inhibitory concentration. [file 4379216.f1.pdf]

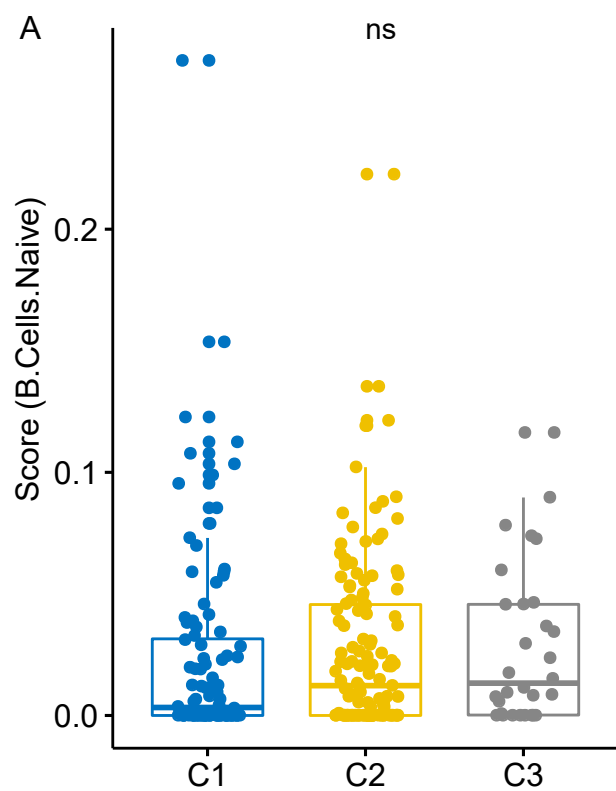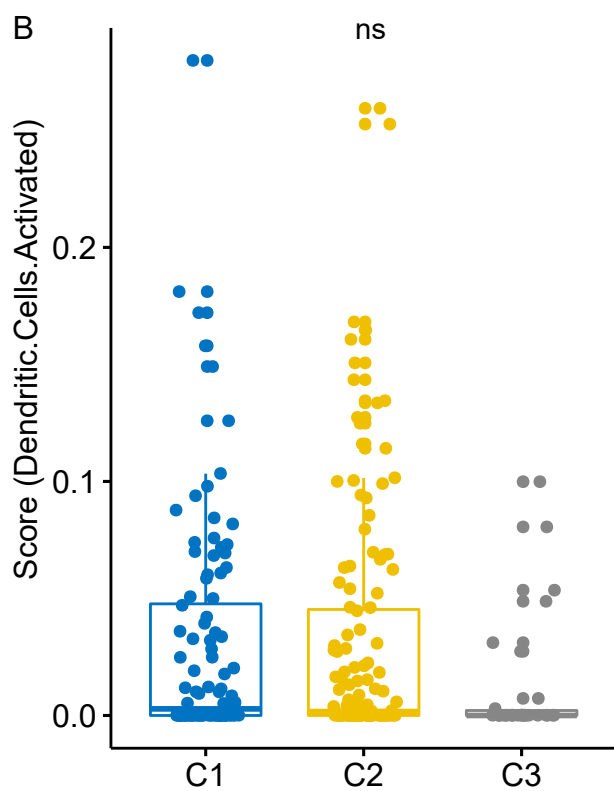

Supplement: Supplementary 3 — Supplementary Figure 3: the scores of naïve B cells and activated dendritic cells between the three groups. Ns: not significant. [file 4379216.f3.pdf]

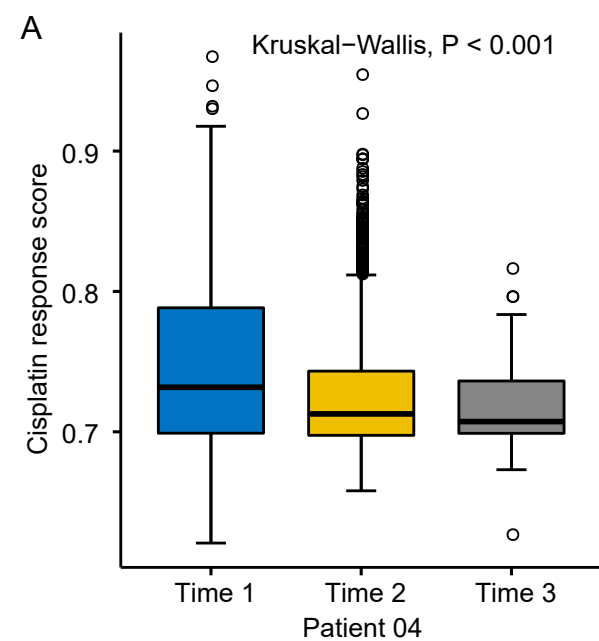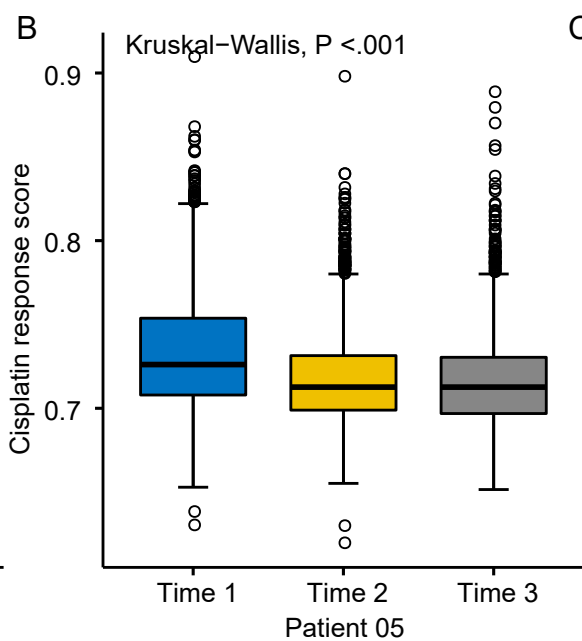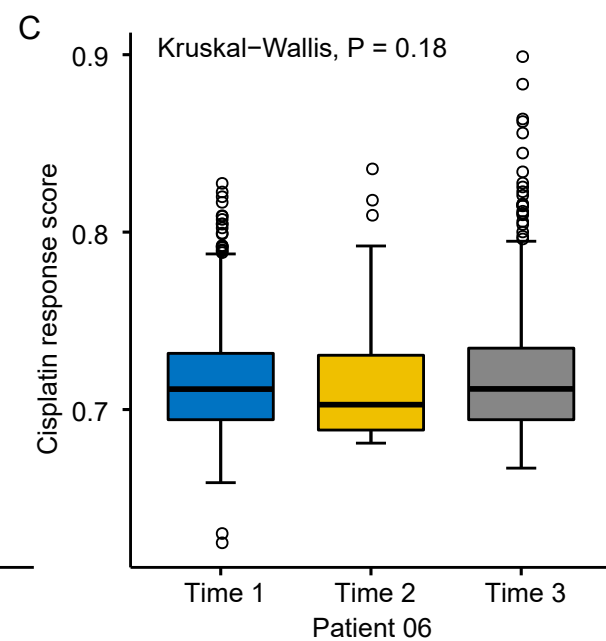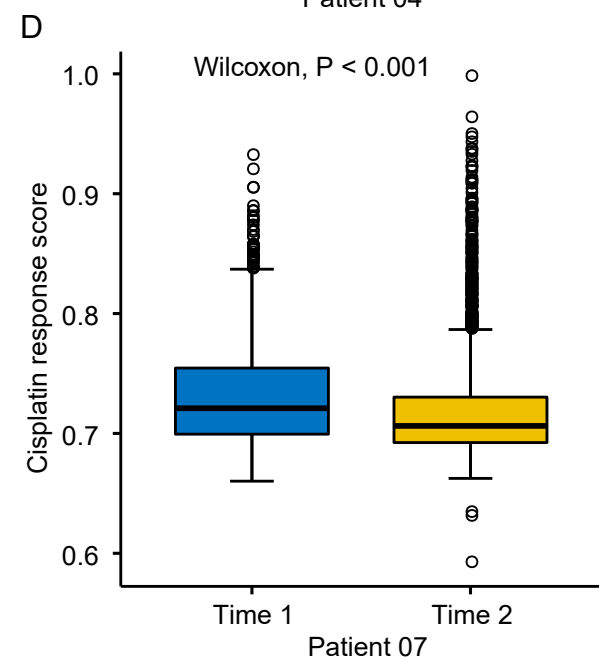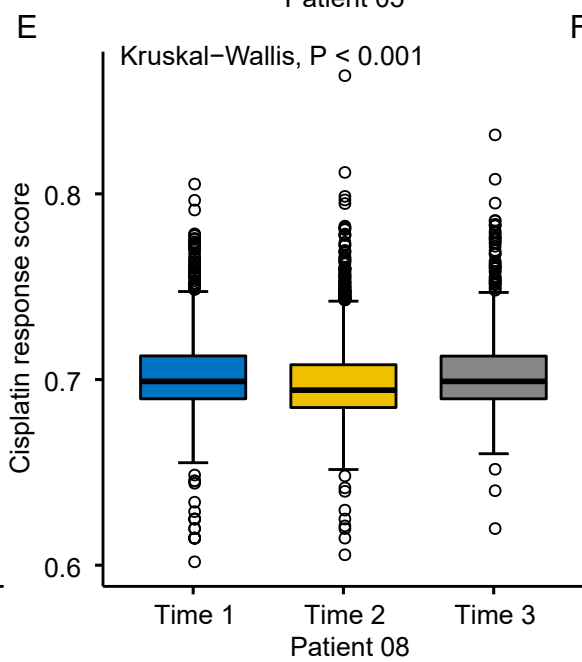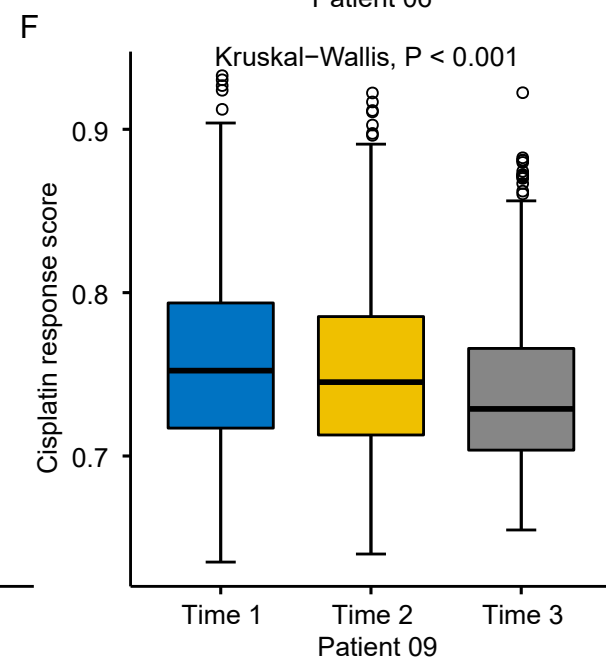

Supplement: Supplementary 4 — Supplementary Figure 4: the cisplatin response scores at three time points for patient 04 (A), patient 05 (B), patient 06 (C), patient 07 (D), patient 08 (E), and patient 09 (F). [file 4379216.f4.pdf]
