# Supplementary material for: Identification of an Immune Gene-Based Cisplatin Response Model and CD27 as a Therapeutic Target against Cisplatin Resistance for Ovarian Cancer
Source: J Immunol Res. 2022 May 18;2022:4379216. doi: 10.1155/2022/4379216 (PMC9133897; doi:10.1155/2022/4379216)
Supplement: Supplementary 5 — Supplementary Table 1: the scores of LM22 cell types between the cisplatin-sensitive (A2780S) and cisplatin-resistant (A2780CP) groups in the GSE23553 dataset. [file 4379216.f5.pdf]

Supplementary table 1. The scores of LM22 cell types between the cisplatin sensitive (A2780S) and resistant (A2780CP) groups in the GSE23553 dataset.

LM22 cell types	Average values		Fold change	P value
	A2780IR	A2780CP		
T cells regulatory Tregs	0.152	0.061	0.400	0.004
B cells naive	0.080	0.146	1.830	0.009
Dendritic cells activated	0.024	0.067	2.779	0.009
Macrophages M1	0.009	0.000	0.029	0.026
NK cells resting	0.020	0.000	0.000	0.028
T cells follicular helper	0.090	0.144	1.597	0.041
Dendritic cells resting	0.012	0.000	0.000	0.074
Plasma cells	0.039	0.021	0.538	0.093
Neutrophils	0.003	0.000	0.000	0.176
T cells CD8	0.312	0.283	0.905	0.310
NK cells activated	0.040	0.076	1.898	0.310
B cells memory	0.019	0.000	0.000	0.405
Mast cells resting	0.018	0.024	1.371	0.630
Macrophages M0	0.003	0.009	2.516	0.655
Monocytes	0.008	0.005	0.613	0.669
Macrophages M2	0.150	0.154	1.022	0.937
T cells CD4 memory resting	0.020	0.011	0.521	1.000
