# Supplementary material for: Identification of an Immune Gene-Based Cisplatin Response Model and CD27 as a Therapeutic Target against Cisplatin Resistance for Ovarian Cancer
Source: J Immunol Res. 2022 May 18;2022:4379216. doi: 10.1155/2022/4379216 (PMC9133897; doi:10.1155/2022/4379216)
Supplement: Supplementary 6 — Supplementary Table 2: eighty-six DEGs between cisplatin-resistant and cisplatin-sensitive groups. [file 4379216.f6.pdf]

Supplementary table 2. Eighty-six DEGs between cisplatin-resistant and -sensitive groups.

Symbol	Average values		Fold change	P value	FDR	Regulation
	Cisplatin sensitive	Cisplatin resistant				
HTR2B	9.85	3.79	0.38	0.00	0.00	Down
TXK	7.75	3.62	0.47	0.00	0.00	Down
HPSE	4.20	5.62	1.34	0.00	0.00	Up
HHEX	4.65	7.14	1.54	0.00	0.00	Up
CD160	5.06	9.81	1.94	0.00	0.00	Up
FZD2	8.35	7.12	0.85	0.00	0.00	Down
QPCT	10.07	5.70	0.57	0.00	0.00	Down
CHST15	5.23	6.25	1.19	0.00	0.05	Up
TRIB2	4.95	6.90	1.39	0.00	0.06	Up
HNMT	5.88	4.15	0.71	0.00	0.09	Down
SPOCK2	7.83	6.69	0.85	0.00	0.17	Down
MXD1	5.68	6.52	1.15	0.00	0.17	Up
EAF2	4.02	4.54	1.13	0.00	0.23	Up
RCAN3	5.52	6.17	1.12	0.00	0.23	Up
DENND5B	5.50	5.13	0.93	0.00	0.23	Down
BRSK2	5.55	5.25	0.95	0.00	0.24	Down
EFNA5	6.17	5.35	0.87	0.00	0.31	Down
SCN9A	5.05	3.21	0.64	0.00	0.38	Down
REPS2	4.16	4.53	1.09	0.00	0.39	Up
DUSP2	6.39	7.16	1.12	0.00	0.46	Up
EGR2	4.42	5.48	1.24	0.00	0.51	Up
ZNF165	4.20	7.20	1.71	0.00	0.51	Up
PDK1	7.66	6.66	0.87	0.00	0.64	Down
ICA1	6.99	5.96	0.85	0.00	0.66	Down
MBL2	4.40	3.51	0.80	0.00	0.90	Down
MAP3K13	5.06	5.50	1.09	0.00	0.92	Up
TNFRSF13B	5.63	5.39	0.96	0.00	0.94	Down
MAN1A1	3.88	5.48	1.41	0.00	0.95	Up
LAMP3	5.23	8.98	1.72	0.00	1.00	Up
TRPM4	5.96	6.43	1.08	0.00	1.00	Up
CXCL10	6.54	5.45	0.83	0.00	1.00	Down
RSAD2	4.58	4.75	1.04	0.00	1.00	Up
ZNF442	3.88	4.19	1.08	0.00	1.00	Up
ST3GAL6	3.65	3.47	0.95	0.00	1.00	Down
PPFIBP1	5.28	6.07	1.15	0.00	1.00	Up
TNIP3	5.73	4.01	0.70	0.00	1.00	Down
RGS1	3.67	3.34	0.91	0.00	1.00	Down
ZNF222	5.90	7.07	1.20	0.00	1.00	Up
ABCB9	6.37	5.92	0.93	0.01	1.00	Down

KYNU	4.99	4.90	0.98	0.01	1.00	Down
BCL7A	6.92	6.37	0.92	0.01	1.00	Down
PASK	6.30	5.81	0.92	0.01	1.00	Down
IGSF6	3.81	3.71	0.97	0.01	1.00	Down
LILRA2	4.91	4.76	0.97	0.01	1.00	Down
STXBP6	3.99	4.20	1.05	0.01	1.00	Up
AZU1	5.57	5.96	1.07	0.01	1.00	Up
CD37	4.50	4.29	0.95	0.01	1.00	Down
GSTT1	6.09	5.77	0.95	0.01	1.00	Down
CHST7	6.38	6.92	1.08	0.01	1.00	Up
TREM1	4.87	5.04	1.04	0.01	1.00	Up
MAP9	6.60	7.03	1.07	0.02	1.00	Up
CD79A	5.55	5.39	0.97	0.02	1.00	Down
PLEKHG3	5.60	5.40	0.97	0.02	1.00	Down
WNT5B	4.42	4.59	1.04	0.02	1.00	Up
KIR2DL1	6.86	6.64	0.97	0.02	1.00	Down
FZD3	8.19	6.94	0.85	0.02	1.00	Down
CHI3L1	4.73	4.60	0.97	0.03	1.00	Down
CXCL11	3.99	4.74	1.19	0.03	1.00	Up
CAMP	6.04	6.28	1.04	0.03	1.00	Up
MZB1	5.32	5.16	0.97	0.03	1.00	Down
PRR5L	4.56	4.47	0.98	0.03	1.00	Down
TMEM255A	3.17	3.06	0.97	0.03	1.00	Down
PTGER2	4.11	3.52	0.86	0.03	1.00	Down
EPB41	5.73	6.24	1.09	0.03	1.00	Up
FCGR3B	4.73	4.53	0.96	0.03	1.00	Down
BIRC3	4.52	4.80	1.06	0.03	1.00	Up
GPR18	3.52	3.32	0.94	0.03	1.00	Down
EPHA1	5.14	5.26	1.02	0.03	1.00	Up
CD27	4.90	5.09	1.04	0.03	1.00	Up
CLC	4.73	4.49	0.95	0.03	1.00	Down
CD1A	5.82	5.56	0.95	0.03	1.00	Down
FBXL8	5.20	4.92	0.95	0.04	1.00	Down
GAL3ST4	6.36	6.12	0.96	0.04	1.00	Down
SMPDL3B	6.20	6.53	1.05	0.04	1.00	Up
TLR8	4.18	4.29	1.03	0.04	1.00	Up
NTN3	5.19	5.05	0.97	0.04	1.00	Down
IFNG	4.99	4.72	0.95	0.04	1.00	Down
SPAG4	6.23	6.90	1.11	0.04	1.00	Up
ALOX15	4.63	4.50	0.97	0.04	1.00	Down
LTC4S	4.71	4.82	1.02	0.04	1.00	Up
CCR3	4.72	4.43	0.94	0.04	1.00	Down
NFE2	5.03	4.38	0.87	0.04	1.00	Down
RRP12	6.37	6.58	1.03	0.05	1.00	Up

GPR183	3.69	3.53	0.96	0.05	1.00	Down
CD5	6.47	6.38	0.99	0.05	1.00	Down
CD247	5.74	4.79	0.83	0.05	1.00	Down
