# Supplementary material for: Identification of an Immune Gene-Based Cisplatin Response Model and CD27 as a Therapeutic Target against Cisplatin Resistance for Ovarian Cancer
Source: J Immunol Res. 2022 May 18;2022:4379216. doi: 10.1155/2022/4379216 (PMC9133897; doi:10.1155/2022/4379216)
Supplement: Supplementary 2 — Supplementary Figure 2: upset plot showing the relationships of 14 immune genes with 22 immune cell types. [file 4379216.f2.pdf]

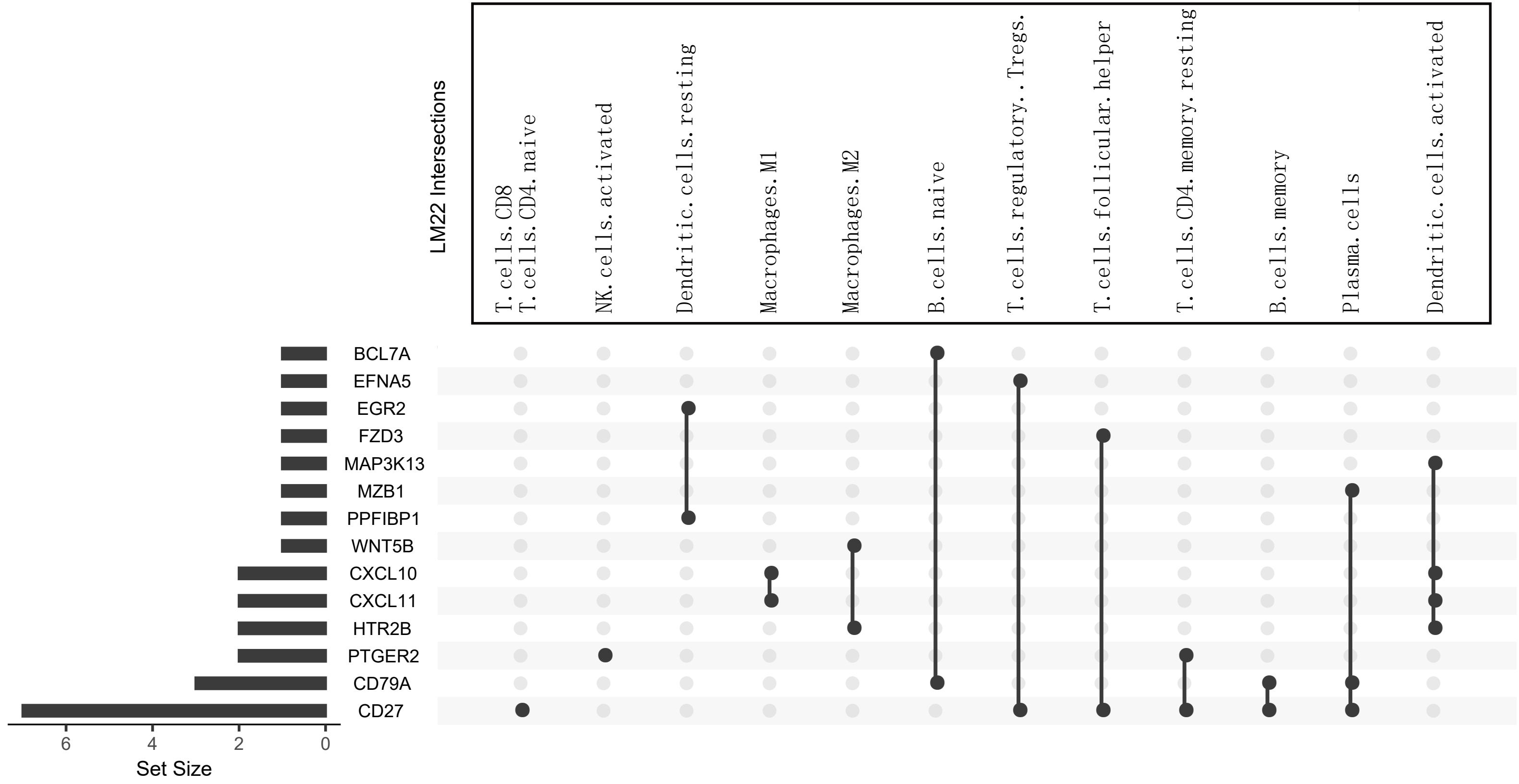

LM22 Intersections
T.cells.CD8
T.cells.CD4.naive
NK.cells.activated
Dendritic.cells.resting
Macrophages.M1
Macrophages.M2
B.cells.naive
T.cells.regulatory..Tregs.
T.cells.follicular.helper
T.cells.CD4.memory.resting
B.cells.memory
Plasma.cells
Dendritic.cells.activated
BCL7A
EFNA5
EGR2
FZD3
MAP3K13
MZB1
PPFIBP1
WNT5B
CXCL10
CXCL11
HTR2B
PTGER2
CD79A
CD27
6
4
2
0
Set Size
